# Change in Environmental Benefits of Urban Land Use and Its Drivers in Chinese Cities, 2000–2010

**DOI:** 10.3390/ijerph13060535

**Published:** 2016-05-26

**Authors:** Xiaoqing Song, Kang-tsung Chang, Liang Yang, Jürgen Scheffran

**Affiliations:** 1 Institute of Land Resources and Urban-Rural Planning, School of Geographical Sciences, Guangzhou University, Guangzhou 510006, China; 2 Department of Geography, National Taiwan University, 1, Roosevelt Road, Section 4, Taipei 106, Taiwan; ktchang@ntu.edu.tw; 3 Institute of Geography, University of Hamburg, Hamburg 20144, Germany; lyang@gshdl.uni-kiel.de (L.Y.); juergen.scheffran@uni-hamburg.de (J.S.)

**Keywords:** urbanization, land use, environmental benefits, sustainability, city, P.R. China

## Abstract

Driven by rising income and urban population growth, China has experienced rapid urban expansion since the 1980s. Urbanization can have positive effects on the urban environment; however, improvement of urban environment quality, especially its divergence between relatively developed and undeveloped cities in China, is currently a rather rudimentary and subjective issue. This study analyzed urban environmental benefits among China’s prefectural cities based on their structure of urban land use in 2000 and 2010. First, we divided 347 prefectural cities into two groups, 81 coastal and capital cities in the relatively developed group (*RD*) and 266 other prefectural cities in the undeveloped group (*RP*). Then, we defined three areas of urban environmental benefits, including green infrastructure, industrial upgrade, and environmental management, and developed an assessment index system. Results showed that all prefectural cities saw improvement in urban environmental quality in 2000–2010. Although the *RD* cities had higher income and more population growth, they had less improvement than the *RP* cities during the same period. We also found that demographic and urban land agglomeration among *RD* cities restrained green infrastructure expansion, making green infrastructure unsuitable as a permanent solution to environmental improvement. It is therefore urgent for China to promote balanced improvement among the three areas of urban environmental benefits and between the *RD* and *RP* cities through regional differentiation policies.

## 1. Introduction

China has experienced rapid urban transformation and expansion since the reform and open-door policy in 1978. It has been estimated that construction land (settlements, industrial/mining sites, and transportation) expanded from 27.2 × 10^4^ km^2^ in 1990 to 32.6 × 10^4^ km^2^ in 2006, a 20% increase [1]. Although the central government has implemented rigorous control on urban land expansion to cope with growing land scarcity [2,3], the expansion has continued unabatedly with an increase of 2.08 × 10^4^ km^2^ urban construction land in 2000–2010 [4]. Many studies have attributed urban land expansion in China to higher income and urban population growth [5,6,7,8,9,10,11]. These observations are supported by government statistics, which show that per capita disposable income of urban households increased by 12,830 yuan in 2000–2010 [12] and urban population in the cities increased by 127.81 million in the same period [13].

Rapid urban expansion in China has been linked to a number of environmental issues, such as farmland loss [14,15,16], soil sealing and pollution [17], air and water pollution [18,19,20], urban heat islands [21,22], and biodiversity loss [23]. More recently, urban environmental quality has become the focus of scientific research and the hot topic among the public in China. Examples include fine particulate (PM_2.5_) in China [18], increased flows of nitrogen and phosphorus in the food system of Beijing [20], and brownfields (sites polluted or potentially polluted by hazardous substances) in China’s major cities [17]. It is generally agreed that substantial socio-economic development and rapid urbanization in the past three decades in China were gained at the cost of environmental quality, both natural and urban. Thus, the central government has begun to pay close attention to searching for a win-win model for socio-economic development and environmental sustainability. Similar efforts have also been initiated by local governments, such as the low carbon eco-city program [24,25].

One aspect of urbanization in China that has not received much attention is its positive effects on the environment (environmental benefits hereafter). The limited literature on the topic has shown that urban land use at the city level can bring about environmental benefits, together with multiple other urban system services, to contribute to human well-being, especially in developing countries. The stage model of urban environmental evolution based on a comparative study in East Asia, e.g., Japan, South Korea, and China, shows urban environmental evolution from poverty, industrial pollution, mass consumption, to eco-city stage caused by rising income and urbanization [26]. A system dynamic simulation model of the drivers and environmental impacts of urban growth suggests an improvement of local environmental quality in Shenzhen, South China, as a result of increasing affluence and economic growth [27]. Thus, our working hypothesis in this study is that the urban environment could improve with rising income and urbanization.

Using the above working hypothesis, the main purpose of this study is to analyze urban environmental benefits among China’s prefectural cities based on their structure of urban land use in 2000 and 2010. Urban land use includes construction land, industrial land, parks, green space, and public infrastructure. We assume that the structure of urban land use changes over time as a response to increasing urban population and affluence, and these changes can in turn affect urban environmental quality. We further assume that the change in environmental benefits varies among cities as cities in China experience different rates of urban expansion and income growth. This second assumption is supported by a study, which reported urban expansion rate and intensity were significantly higher in the eastern region prior to 2000 but the trend reversed after 2000 [28]. The same study also justifies our selection of the study period 2000–2010, as it represents an important period of change in China’s urbanization.

One of the problems of analyzing the relationship between urbanization and the environment is the definition of environmental quality [29,30]. As pointed out by Nichol and Wong [31], environmental quality is an abstract concept resulting from both human and natural factors operating at different spatial scales. This is why studies have chosen different indicators to define the concept. For example, in their study of the relationship between urbanization and the environment in the Huai River Basin in China, Guo, Wang, Nijkamp, and Xu [32] first defined the quality of environment as consisting of the three functional groups of environmental endowment, environmental pressure, and environmental management, and then used a variety of indicators to measure each of the functional groups.

Based on a literature review, this study defines the environmental benefits of urban land use as the positive effects on the environment posed by urban land use activities, which consist of the following three areas. The first is environmental improvement through expansion of green infrastructure such as greenways, parks, and other conservation lands [23,33,34]. As urban residents grow in number and become wealthier, they are likely to ask for more green spaces, which have shown to provide a range of benefits such as increasing home sale price [35,36], improving urban quality of life and public health [37,38], and even helping cities to adapt to climate change [39].

The second is environmental improvement through conversion of manufacturing to service industry. As urban land expands as a result of population growth and rising income [9,40], land scarcity is bound to increase land prices and to encourage conversion of urban land from manufacturing to more valuable service industry [27,41,42]. This, in turn, can improve the environmental quality of a city through, for example, reduction of pollutants.

The third is environmental improvement through better environmental management [43,44]. Municipal solid waste management has become an important issue in China with rapid urbanization [45]. Safe waste disposal rates vary among regions, with more affluent cities in the coastal region having higher rates than the central region [46]. Meanwhile, urban residents expect to have access to water and sanitation services, which can improve with higher income [47] and environmental governance [48]. 

Urban land use as a link between the environment and urban residents plays a key role in environmental change in the cities. An in-depth understanding of the environmental benefits of urban land use is therefore important for achieving well-balanced and sustainable urban development in China. Although this study focuses on the environmental benefits of urban land use, it also provides an opportunity to look into problems hidden in China’s rapid urbanization, which deserve consideration in government policies. 

Following the introduction, Section 2 describes the research method, including the study sites, assessment of environmental benefits of urban land use, and a weighted regression model to explore the driving factors for changes in environmental benefits. Section 3 presents the patterns and changes of environmental benefits of urban land use in 2000 and 2010 and the results of the regression analysis. Section 4 discusses the relationship between income, urban population, and environmental benefits and the implications of sustainable urban land use. The paper closes with a summary and recommendations for policy changes.

## 2. Materials and Methods

### 2.1. Prefectural Cities

**Figure 1:**
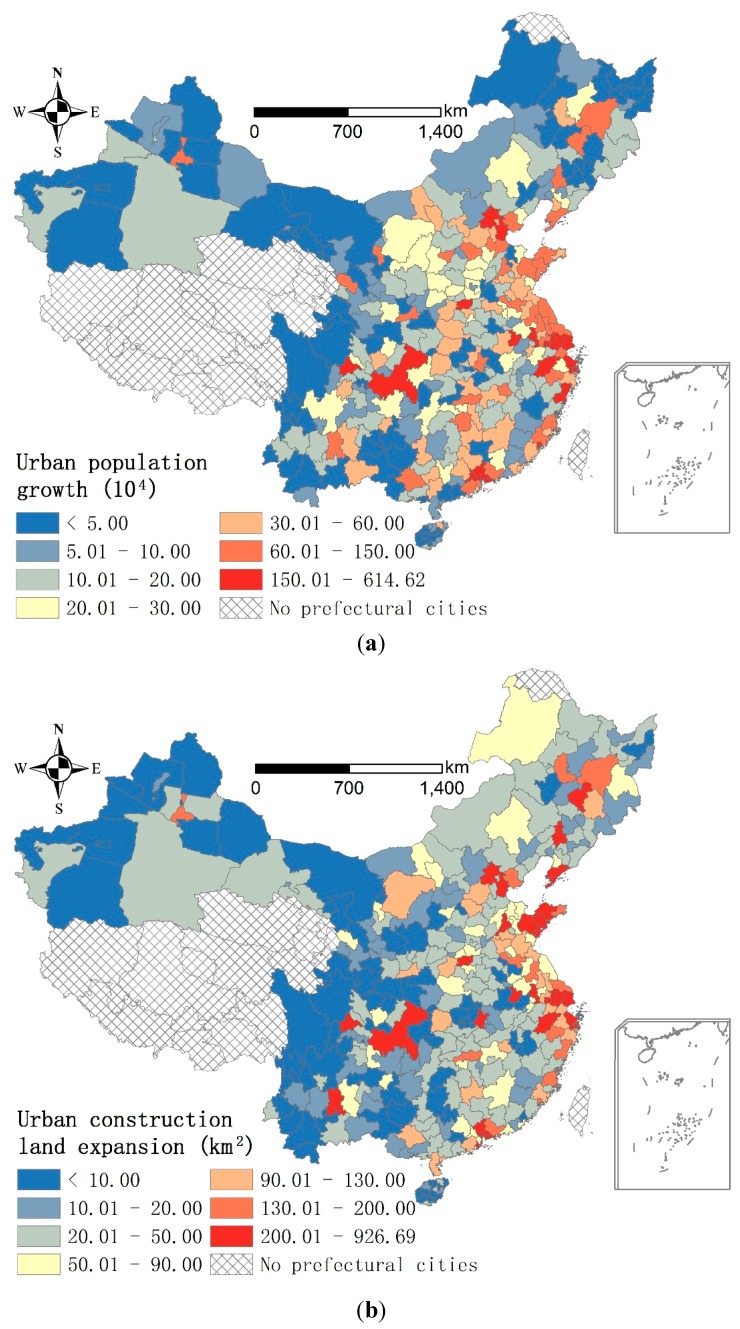
Urban population growth and urban construction land expansion in the prefectural cities, China, 2000–2010. (**a**) Urban population growth (10^4^); (**b**) Urban construction land expansion (km^2^).

**Figure 2:**
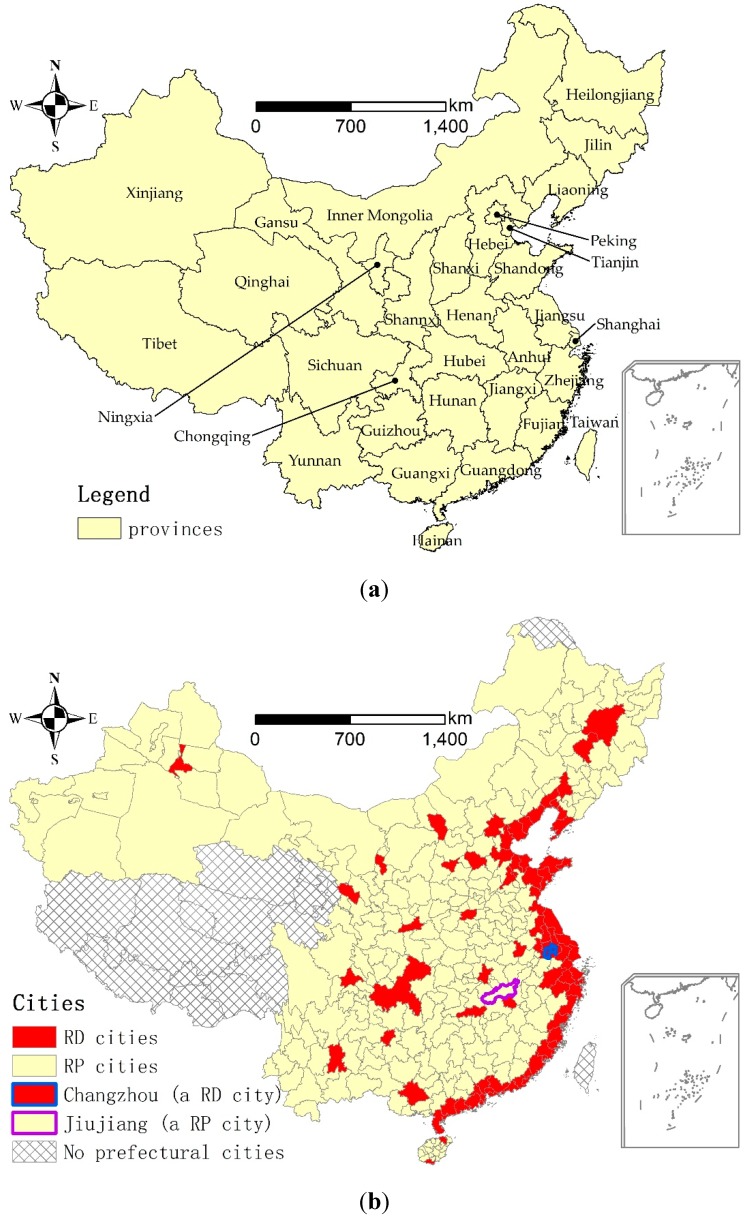
Provinces and the prefectural cities in China. (**a**) Provinces in China; (**b**) The prefectural cities in China.

A prefectural city is an administrative division, ranking below a province and above a county in China’s administrative structure. A prefectural city comprises urban and rural areas. This study only considered the urban areas, which are named cities or city districts. Similar to the regional disparity [8], urban expansion also varies among China’s prefectural cities. As shown in Figure 1, both urban population growth and construction land expansion in 2000–2010 were higher in the coastal cities and capital cities (provincial capitals and provincial-level municipalities of Beijing, Tianjin, Shanghai, and Chongquing) than other prefectural cities. Although per capita disposable income of urban households in all of the prefectural cities increased dramatically in 2000–2010, it was 21,377 yuan in coastal and capital cities and only 15,326 yuan in other prefectural cities in 2010 [49]. Considering the remarkable differences in urbanization and income growth, we grouped the 81 coastal and capital cities into relatively developed cities (*RD* cities) and the rest of the prefectural cities (266) into *RP* cities (Figure 2). This subdivision of prefectural cities provided the basis for subsequent data analysis on: (1) the spatial distribution of environmental benefits among the prefectural cities in 2000 and 2010; (2) the change of environmental benefits between the *RD* and *RP* cities in 2000–2010; and (3) the driving factors influencing the change of environmental benefits in 2000–2010.

### 2.2. Assessment of Environmental Benefits of Urban Land Use

As indicated previously, we expected environmental benefits of urban land use to derive from green infrastructure, industrial upgrade, and better environmental management. Based on a literature review, we selected specific indicators for these three areas of environmental benefits for subsequent data analysis.

Green infrastructure has been introduced as a planning tool for promoting human health and ecosystem services in the cities [50,51], which in turn are closely related to not just the amount but also the pattern of green infrastructure [52,53,54]. In this study, area of parks and green space per capita, density of parks and green space, and park size were selected to represent the total amount, the allocation, and the pattern of green infrastructure, respectively, in the cities [52,55,56,57,58,59]. 

Industrial upgrade can reduce pollutants in the cities such as industrial SO_2_ and industrial wastewater; therefore, SO_2_ emission and discharge and reuse of industrial waste water are important indicators for revealing the environmental dimension of sustainable urbanization [42,55,57,58,60]. Additionally, amount of SO_2_ per unit of urban area has been used for measuring urban environmental competitiveness [56]. In this study, we used the indicators of density of industrial land, density of industrial SO_2_ discharged, and rate of discharged industrial wastewater meeting national standard to measure the environmental benefits related to industrial upgrade.

To measure the quality of environmental management, we used the indicators of proportion of and intensity of urban maintenance and construction fund invested in environmental expenditure, and the disposal rate of domestic harmless garbage [42,55,57,58,59,60,61]. Table 1 shows the indicators selected for this study, using the years of 2000 and 2010.

Of the nine indicators, only density of industrial land and density of industrial SO_2_ discharged were expected to be negatively related to the environmental benefits [62]; therefore, to be consistent, the reciprocals of these two indicators were used in subsequent analysis. Further, all the indicators were normalized to uniform dimension. Then, the value of environmental benefits of urban land use (*ebu*) was calculated using the entropy-based weight method, which ensured that a greater weight was assigned to an indicator with a smaller degree of uncertainty or smaller entropy [63]:

(1) $$ebu=\sum w_{i}\times X_{i}$$

(2) $$w_{i}=\left(1-e_{i}\right)/\sum \left(1-e_{i}\right)$$

(3) $$e_{i}=-\frac{1}{\text{ln}\;n}\sum \left(X_{i}\times \text{ln}X_{i}\right)$$

where *w_i_* is the entropy weight of indicator *i*; *X_i_* is the normalized value of indicator *i*; *e_i_* is the entropy value of indicator *i*; *n* is the number of objects assessed. 

Because *ebu* consisted of three aspects of environmental benefits, it could be divided into *ebuei* for green infrastructure, *ebudi* for industrial upgrade, and *ebuma* for environmental management. This subdivision allowed an examination of the three aspects separately in the results.

### 2.3. Regression Model Specification

To explore the driving factors for change of environmental benefits, this study employed a weighted regression model, using *ebu* as the dependent variable. The independent variables included five socio-economic and demographic variables and one interaction variable.

Per capita disposable income of urban households (*pci*) and size of urban population (*aup*) were expected to be positively related to improvement of *ebu* [26,27]. Driven by higher income, rapid urbanization in China represents a demographic transfer from rural areas to cities or from *RP* to *RD* cities. In other words, *aup* was expected to be conditional to *pci*, *i.e.*, a city with a higher income level is expected to have a larger urban population and vice versa. Therefore, we introduced an interaction variable between *aup* and *pci* in the model. Land urbanization (*lu*) and demographic urbanization (*du*) characterizing the process of rapid urbanization in China were also included [64]. Additionally, as explained in the introduction section, change in *ebu* was conditional to urban land scarcity; thus, per capita urban construction land area (*pcucl*) was introduced as an independent variable to measure urban land scarcity. Table 2 lists these independent variables.

In equation form, the econometric model can be expressed as follows:

(4) $$ln\left(y_{j}\right)=c+\beta ln\left(x_{j}\right)+\gamma ln\left(pci_{j}\right)\times ln\left(aup_{j}\right)+\varepsilon_{j}$$

where *y_j_* is the dependent variable or *ebu* of prefectural city *j*; *x_j_* is a vector of the independent variables; $ln\left(pci_{j}\right)\times ln\left(aup_{j}\right)$ is the interaction variable; *c* is a common intercept; *β* and *γ* denote unknown parameters to be estimated; and *ε_j_* is an unobserved error term.

The model was computed for 2000 and 2010 using the software EViews 7.2 (Quantitative Micro Software, Irvine, CA, USA). The unknown parameters were estimated using the weighted least squares regression method, which is designed to maximize the efficiency of parameter estimation. We explored the mechanism of changes in *ebu* by analyzing changes in these estimated parameters for 2000 and 2010.

## 3. Results

### 3.1. Spatial Pattern of Environmental Benefits of Urban Land Use

**Figure 3:**
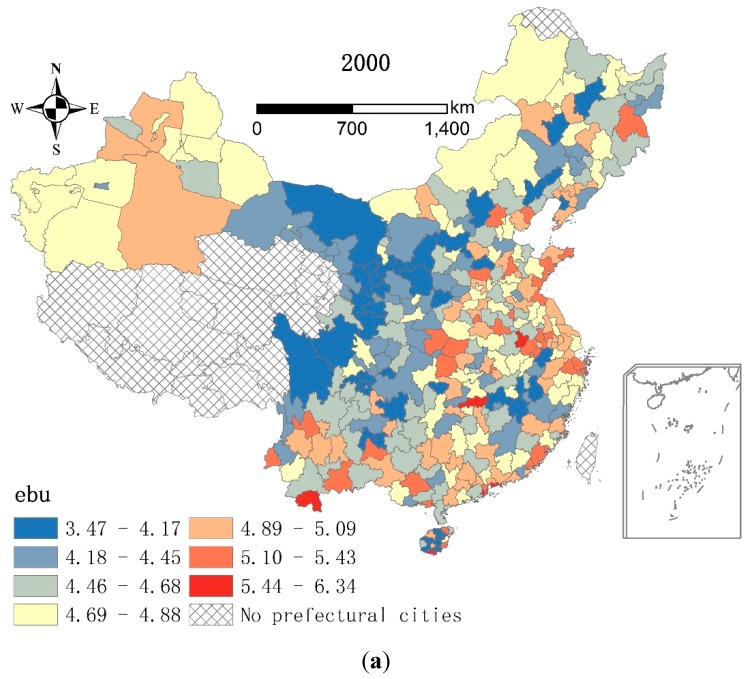

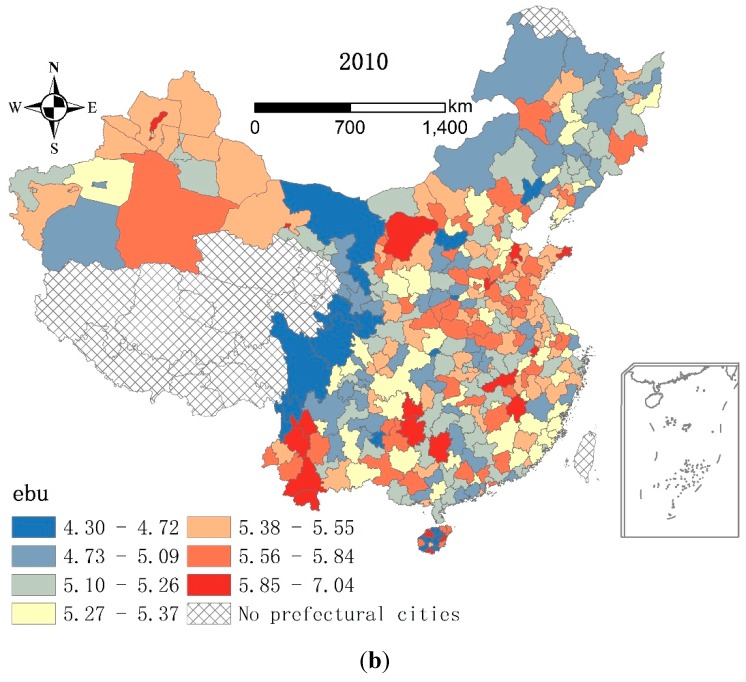
Environmental benefits of urban land use in the prefectural cities, China, 2000 and 2010. (**a**) Environmental benefits of urban land use in the prefectural cities, China, 2000; (**b**) Environmental benefits of urban land use in the prefectural cities, China, 2010.

Figure 3 shows the environmental benefits of urban land use at the city level in 2000 and 2010. In 2000, the *ebu* values range from 3.47 to 6.34, with an average of 4.68. The average *ebu* among the *RD* cities is 4.94, as most coastal and capital cities have *ebu* values above 4.69. Meanwhile, the average *ebu* among the *RP* cities is 4.60, as most cities in Sichuan, Gansu, Shaanxi, Ningxia, Jiangxi, and the western Inner Mongolia have *ebu* values below 4.45. In general, more environmental benefits of urban land use are found among the *RD* cities than the *RP* cities.

In 2010, the *ebu* values range from 4.30 to 7.04, with an average of 5.32 (Figure 3). In a different pattern from 2000, most cities with above-average *ebu* values are *RP* cities. Although *RP* cities in western Sichuan, central and southern Gansu, and western Inner Mongolia have much lower *ebu* values, the proportion of *RP* cities with *ebu* values higher than 5.56 amounts to 76%. The *RD* cities have an average *ebu* value of 5.36, as 77.78% of these cities have *ebu* values lower than 5.55. In general, most *RP* cities have more environmental benefits than the *RD* cities in 2010.

### 3.2. Change in Environmental Benefits of Urban Land Use

**Figure 4:**
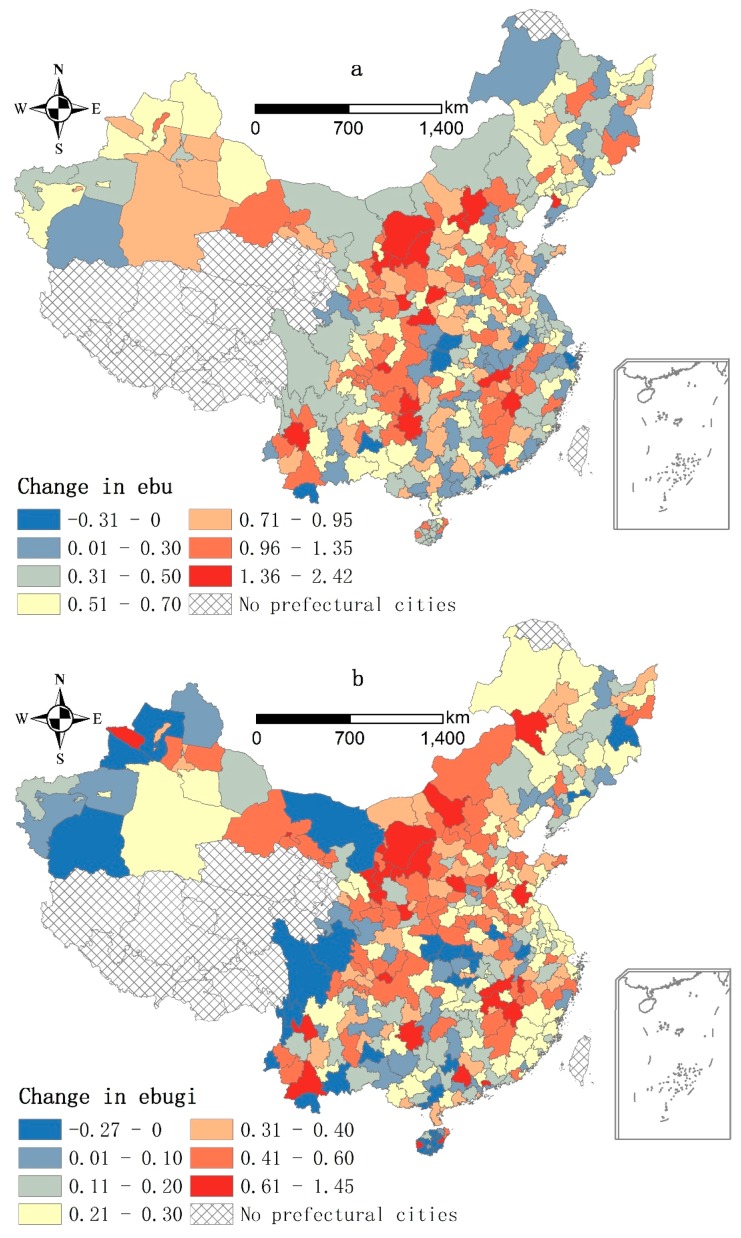

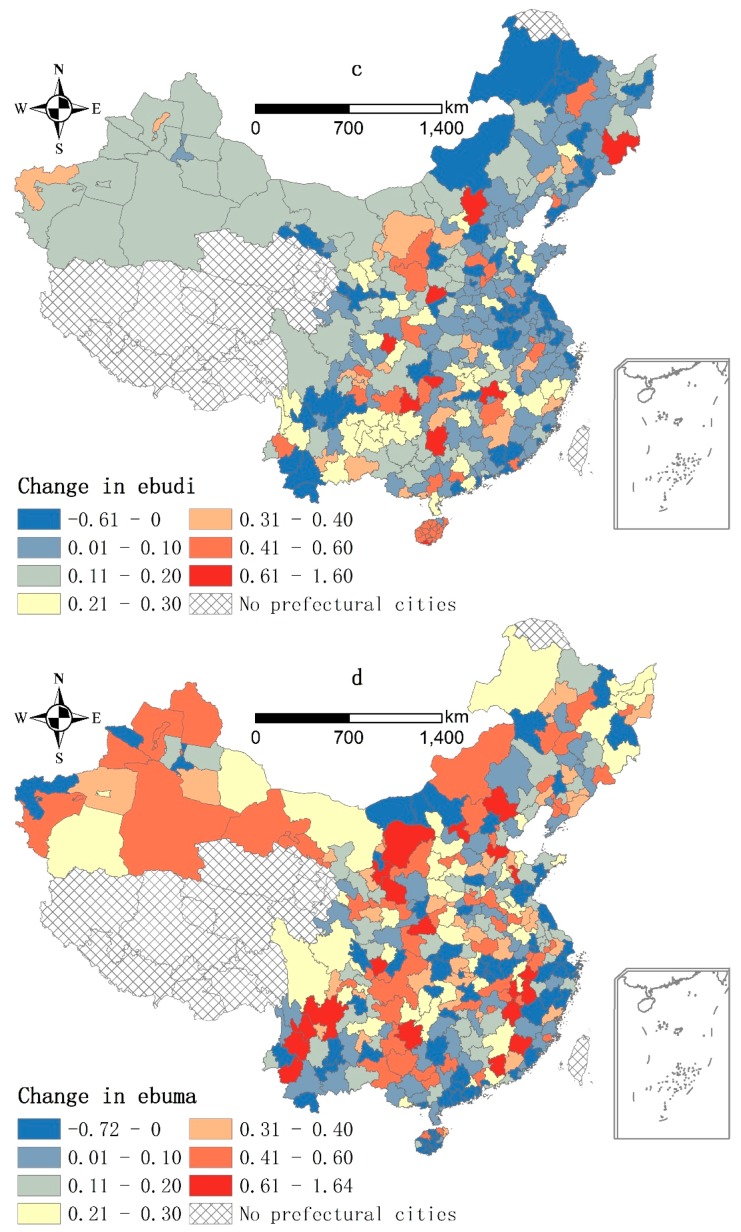
Change in environmental benefits of urban land use in the prefectural cities, China, 2000–2010. (**a**) Change in *ebu* (environmental benefits of urban land use) in the prefectural cities, China, 2000–2010; (**b**) Change in *ebugi* (environmental benefits of green infrastructure) in the prefectural cities, China, 2000–2010; (**c**) Change in *ebudi* (environmental benefits from industrial upgrade) in the prefectural cities, China, 2000–2010; (**d**) Change in *ebuma* (environmental benefits from environmental management) in the prefectural cities, China, 2000–2010.

Change of *ebu* was mapped by subtracting the *ebu* in 2000 from the *ebu* in 2010. As shown in Figure 4a, changes in *ebu* at the city level range from −0.31 to 2.42, with 97% of the cities having increases. When examined in detail, there are differences between the *RD* and *RP* cities. Most *RD* cities have change values between −0.31 and 0.70. Meanwhile, cities with increases of more than 0.71 are primarily *RP* cities in Jiangxi, western Yunnan, eastern Guizhou, eastern Sichuan, northern Shaanxi, northern and eastern Gansu, northern Hebei, and southern Inner Mongolia. In general, the average increase of *ebu* among the *RD* cities is much lower than the *RP* cities between 2000 and 2010, as shown in Table 3.

Figure 4b–d show changes of *ebugi*, *ebudi*, and *ebuma*, the three aspects of environmental benefits, separately. The three maps are based on the same class breaks of 0, 0.1, 0.2, 0.3, 0.4, and 0.6. The spatial pattern of changes in *ebugi* in Figure 4b is similar to that of *ebu* in Figure 4a, with higher increases (>0.3) mainly among the *RP* cities. Most *RD* cities, on the other hand, have values between 0.21 and 0.30. Figure 4c shows that changes in *ebudi* are less than 0.30 in most cities, with values generally between −0.60 and 0.10 for *RD* cities and generally greater than 0.11 for *RP* cities. Figure 4d shows that changes of *ebuma* exceed 0.11 in most cities. Cities, which have values higher than 0.21, are primarily *RP* cities. Meanwhile, *RD* cities have lower increases in *ebuma* overall. In fact, a number of *RD* cites along the coast have decreases in *ebuma*.

To further clarify the differences between the *RD* and *RP* cities, we calculated the contribution breakdown of *ebugi*, *ebudi*, and *ebuma* to *ebu* among the whole prefectural cities, *RD* cities, and *RP* cities, as shown in Table 3. For the whole prefectural cities, contribution of *ebugi* to *ebu* is greatest followed by *ebuma*, whereas *ebudi* has the lowest contribution. Contribution of *ebugi* is higher among the *RD* cities than the *RP* cities. On the other hand, contributions of *ebudi* and *ebuma* among the *RP* cities are greater than those of the *RD* cities.

### 3.3. Example: Environmental Benefits of Urban Land Use in Changzhou and Jiujiang

We selected Changzhou and Jiujiang to illustrate the differences in the change in environmental benefits of urban land use between a *RD* city and a *RP* city. Figure 2 shows the location of these two cities. Changzhou is a *RD* city located in Jiangsu province, with 1.33 million of urban population growth and 92.49 km^2^ of urban land expansion in 2000–2010. The per capita disposable income of urban households in Changzhou increased from 8540 yuan to 25,875 yuan. Jiujiang is a *RP* city located in the northern Jiangxi province, with 0.15 million of urban population growth and 46.77 km^2^ of urban land expansion in 2000–2010. The per capita disposable income of urban households in Jiujiang increased from 5081 yuan to 15,764 yuan.

Table 4 shows that Changzhou’s *ebu* is higher in 2000 but lower in 2010 than Jiujiang’s, as the increase of *ebu* is much less in Changzhou than in Jiujiang. In fact, the increases of the three *ebu* components are all lower in Changzhou than in Jiujiang. In terms of their contribution, *ebugi* is the largest, followed by *ebuma* and *ebudi*, for both Changzhou and Jiujiang. However, Changzhou has a higher contribution of *ebugi* and lower contributions of *ebudi* and *ebuma* than Jiujiang. Table 5 shows the original values of indicators for the *ebu* assessment.

### 3.4. Regression Results

Table 6 lists the statistical description of the independent variables in logarithm. Table 7 shows the regression results. The relationships between *ebu* and *pci*, *aup* and *du* are all positive in 2000. Based on the regression coefficient, *pci* is the most important among the three independent variables, followed by *aup*. The interaction variable of *pci* and *aup* has a low and negative impact on *ebu*. Likewise, *pcucl* and *lu* also have low and negative impacts on *ebu*.

In 2000–2010, the coefficient of *aup* increases by 0.227, which is the largest among all coefficients. Next is the coefficient of *pci*, which has an increase of 0.052. This implies that *aup* and *pci* have more positive impacts on *ebu* in 2010 than in 2000. At the same time, the negative impact of the interaction variable of *pci* and *aup* becomes stronger in 2010. The coefficient of *du* decreases by 0.002 in 2000–2010; this suggests that its impact on *ebu* has no obvious change. It is noted that the impact of *pcucl* turns from negative to positive in 2000–2010, and the positive impact is more significant than the negative impact. The coefficients of *lu* decreases from −0.002 to −0.009 in 2000–2010, indicating a more significant negative impact in 2010.

## 4. Discussion

### 4.1. Relationship between Income, Urban Population Growth, and Environmental Benefits

The literature indicates that environmental benefits of urban land use are positively related to income and urban population growth [26,27]. The increase of *ebu* (Figure 4a) and the increase of the positive coefficients of *pci* and *aup* (Table 7) reveal that growth of per capita disposable income of urban household and urban population indeed brought about the improvement of environmental benefits in the prefectural cities in 2000–2010. However, this inference seems to be contradicted by two observations: the lower average increase of environmental benefits in *RD* than *RP* cities, and the intensifying negative impact of the interaction variable of *pci* and *aup* (Table 7). But this is not the case upon closer examination. The interaction of *pci* and *aup* represents a demographic transfer, driven by higher income, from rural areas to cities or from *RP* to *RD* cities. Rural labor transfer amounted to nearly 390 million in 1978–2009 [65]. Although the arrival of the Lewis turning point in China, a point at which an excess supply of labor is changed to one of labor shortage, has been subject to debate [66,67,68], transferred rural labor actually increased from 128.91 million to 171.67 million in 2000–2009 [69]. This indicates that a large amount of demographic agglomeration in *RD* cities suppressed the improvement of urban environmental benefits in 2000–2010, as shown by the higher negative coefficient of the interaction variable between *pci* and *aup* in 2010.

**Figure 5:**
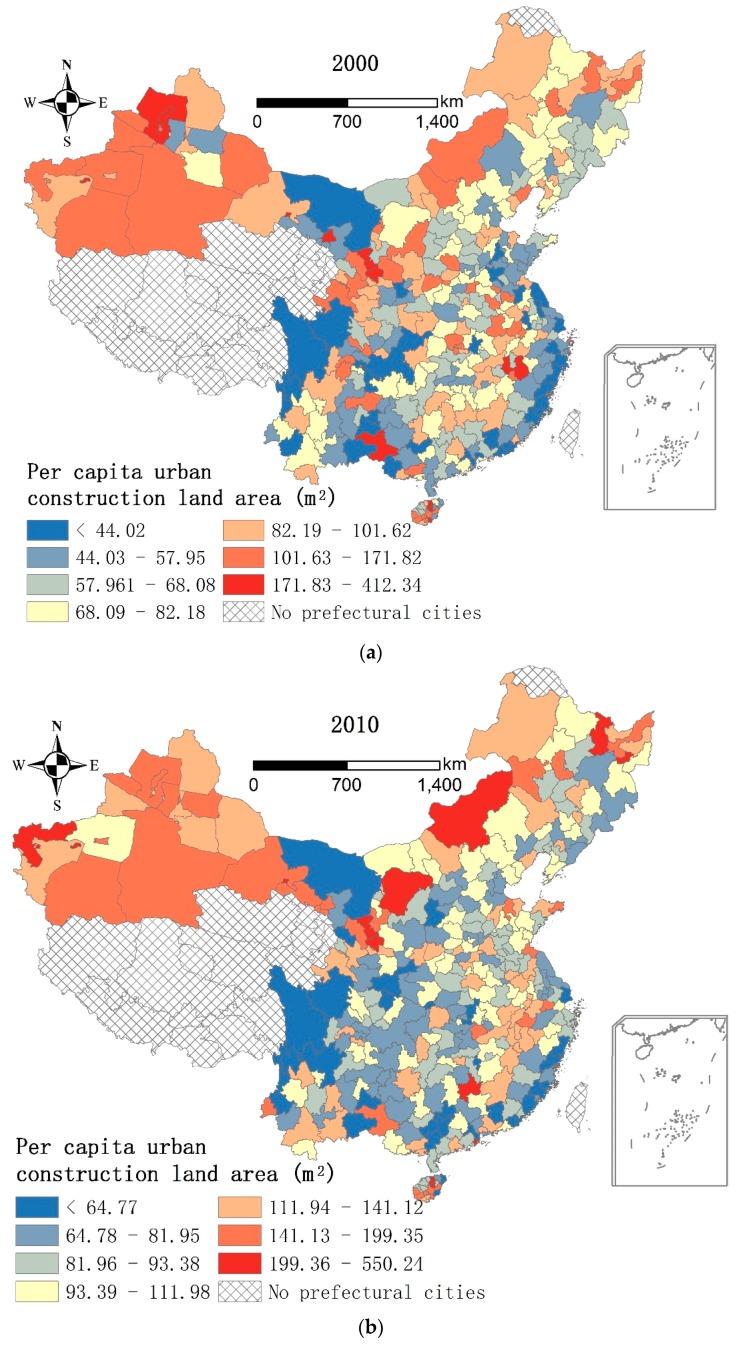
Per capita urban construction land area in the prefectural cities, China, 2000 and 2010. (**a**) Per capita urban construction land area in the prefectural cities, China, 2000; (**b**) Per capita urban construction land area in the prefectural cities, China, 2010.

Demographic agglomeration can have a negative impact on environmental improvement because it causes greater land scarcity and lower *pcucl*. Figure 5 shows that the *RD* cities had a much lower *pcucl* than the *RP* cities in 2000 and 2010. As shown by the original data from *China Urban Construction Statistical Yearbook*, the average *pcucl* of the *RD* cities was 25.76 m^2^ and 18.77 m^2^ lower than the *RP* cities in 2000 and 2010, respectively. Land economics theory indicates that land is apt to be allocated to more valuable uses as land scarcity increases [41]. Thus, as land scarcity worsened in *RD* cities, they restrained green infrastructure expansion for more valuable uses, such as industrial land. For example, per capita area of and density of parks and green space in the *RD* cities increased by 16.1 m^2^ and 9.57% in 2000–2010, which were 1 m^2^ and 3.58% lower than the *RP* cities, respectively. Meanwhile, park size in the *RD* cities increased by 6.56 m^2^, which was 4.52 m^2^ lower than the RP cities. Thus, the average increase of the *ebugi* was much lower in the *RD* cities than the *RP* cities in 2000–2010 (Table 4).

However, previous studies have also revealed that land scarcity can lead to industrial upgrade towards a service economy [27,41,42], which can be beneficial for environmental improvement. Then, if land scarcity becomes more serious, it could bring about more environmental benefits relating to industrial upgrade, especially among *RD* cities. As shown in Figure 4c, *ebudi* values indeed increased in most cities, but increment was lower among *RD* cities. Specifically, the average increase of *ebudi* in the *RD* cities was 0.071 in 2000–2010, which was 0.105 lower than the *RP* cities. This implies that the effects of a service economy on urban environmental improvement have not fully manifested as anticipated. There is much potential to promote industrial upgrade towards a service economy especially among *RD* cities.

According to the analysis above, a lower *pcucl* or more serious land scarcity per se constrained green infrastructure expansion, especially among *RD* cities. Could the opposite be true, *i.e.*, more environmental benefits realized as a result of a higher *pcucl*? In 2000, the *RD* cities had higher *ebu* values (Figure 3) and much lower *pcucl* values (Figure 5) than the *RP* cities overall. This mismatching resulted in the negative impact of *pcucl* on *ebu* in 2000 (Table 7). However, the pattern changed in 2010. Although *ebu* and the *pcucl* in most cities increased in 2000–2010, the *RP* cities had overall higher *ebu* values and also higher *pcucl* values than the *RD* cities. This matching led to the positive impact of *pcucl* on *ebu* in 2010. Additionally, the coefficient of *pcucl* increased from −0.008 to 0.020 in 2000–2010 (Table 4), suggesting that *pcucl* had a stronger impact on *ebu* in 2010 than 2000. In short, if we did not consider the effect of the mismatching, an increase of *pcucl*, which could be interpreted as an increase of urban land supply, could indeed contribute to an improvement of *ebu*.

**Figure 6:**
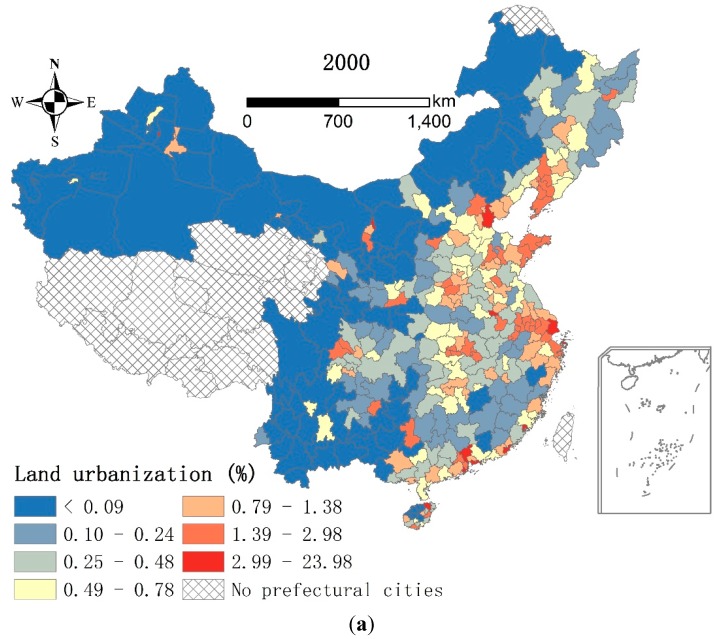

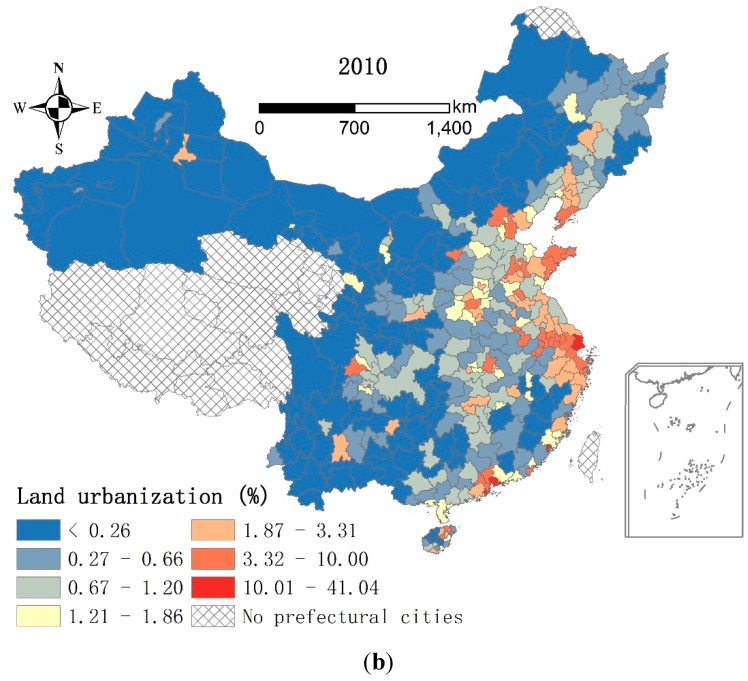
Land urbanization in the prefectural cities, China, 2000 and 2010. (**a**) Land urbanization in the prefectural cities, China, 2000; (**b**) Land urbanization in the prefectural cities, China, 2010.

Since land scarcity due to a large amount of demographic agglomeration can suppress the improvement of *ebu*, it seems reasonable to expand urban land especially in *RD* cities. However, Table 7 shows that land urbanization exerts negative impacts on improvement of *ebu*. To explain this contradiction, we can start with Figure 6, which shows that urban land agglomerated in *RD* cities. Specifically, high *lu* values (>1.39) were found in *RD* cities while most *RP* cities had low *lu* values (<0.78) in 2000. This divergence of the *lu* values was much more apparent than that of the *ebu* values at the city level. Although the *lu* values generally increased in 2010, their spatial pattern at the city level, unlike that of *ebu*, had no obvious changes. The differences between the patterns of *lu* and *ebu* therefore resulted in the negative impacts of *lu* on *ebu* in 2000 and 2010. As high *ebu* values moved towards *RP* cities, high *lu* values moved towards *RD* cities in 2000–2010. Meanwhile, the coefficient of *lu* decreased from −0.002 to −0.009 in 2000–2010 (Table 7), implying that the impact of *lu* had deepened in 2010. Thus, urban land agglomeration in *RD* cities inhibited the improvement of *ebu* among the whole prefectural cities.

### 4.2. Implications for Sustainable Urban Land Use

Although this study focuses on the environmental benefits of urban land use, we have found three aspects of unsustainable urban land use, which deserve consideration in government policies. The first is related to green infrastructure expansion. According to the original data from *China Urban Construction Statistical Yearbook*, for all prefectural cities, the area and the density of parks and green space increased by 1 million hm^2^ and 11.67% in 2000–2010, respectively. Meanwhile, the area of parks and green space per capita and the park size increased by 15.71 m^2^ and 2.23 hm^2^, respectively. The expansion of these green infrastructure indicators indeed resulted in major environmental improvement, which in turn contributed to most improvement in environmental benefits, as shown in Figure 4b and Table 4. Industrial upgrade, however, had the least contribution. Moreover, the *RD* cities had a much higher contribution of green infrastructure expansion, and a much lower contribution of industrial upgrade to the increase of environmental benefits, than the *RP* cities. This finding is consistent with previous studies, which have suggested that green infrastructure expansion is a stage in the process of urbanization forced by income and urban population growth [35,36,37,38,39]. Our previous discussion has also indicated the contribution of higher *pcucl*, *i.e.*, higher urban land supply, to the improvement of environmental benefits. The growing land scarcity, however, has been the key issue of economic development and urbanization in China [3]. In response, urban land supply will be restrained rigorously by a restriction of 120 million hectares for cultivated land to ensure food security [70]. Additionally, according to the Ministry of Land and Resources of the People’s Republic of China, urban growth boundaries of 14 *RD* cities including Peking, Shanghai, Guangzhou, Shenzhen, Chengdu, Wuhan, Zhengzhou, Nanjing, Suzhou, Shenyang, Hangzhou, Xiamen, Xi’an and Guiyang, will be designated in 2015. Furthermore, designation of urban growth boundaries will be extended to 600 cities in China. Thus, the continuing massive increase in urban land supply is not possible in the long run. Further, green infrastructure expansion through increasing urban land supply is not a permanent solution to environmental improvement.

The second is related to the demographic agglomeration in *RD* cities. Although rapid industrialization in *RD* cities contributed to large increases in income, it also led to massive in-migration, which in turn suppressed environmental improvement due to the restrained green infrastructure expansion and the poor performance of industrial upgrade and environmental management [16,65,69,71]. This implies that *RD* cities paid more attention to industrialization than environmental improvement. For example, the proportion and the intensity of urban maintenance and construction fund invested in environmental expenditure increased by 1.28 percent and 7.97 thousand yuan per hm^2^ in 2000–2010 in the *RD* cities, which were 2.32 percent and 14.72 thousand yuan per hm^2^ lower than the *RP* cities. This finding is consistent with the research results of Lu *et al.* [72], which showed that Guangdong Province paid more attention to industrial development and less to living conditions in cities during the period 2000–2010. On the basis of the 2005 Population Survey, Knight, Deng and Li [71] predicted another 123 million migrants moving into cities in China from 2010 to 2020. These migrants will cause more demographic agglomeration in *RD* cities. It is therefore plausible that suppression of environmental improvement will be further intensified if *RD* cities continue to sacrifice environmental improvement for industrialization.

The third is related to urban land agglomeration in *RD* cities [73,74]. To accelerate the reform of the open-door policy in 1978, the Chinese central government implemented unbalanced development strategies to encourage development of *RD* cities ahead of *RP* cities after the mid-1980s. Since then, *RD* cities have been given priority for urban land expansion. Although the central government started to promote regional coordinated development after 2000, *RD* cities still had the priority to attain urban land quota in land use planning. As discussed previously, urban land agglomeration in *RD* cities, however, did not yield more environmental benefits from green infrastructure expansion than in *RP* cities. Urban land scarcity in *RD* cities in fact promoted land conversion to more valuable uses such as industrial use rather than green infrastructure. In other words, urban land agglomeration meant more industrial development and more demographic agglomeration in *RD* cities in a pattern of positive feedback. In turn, land scarcity became more serious, resulting in further restraint of green infrastructure expansion. In short, urban land agglomeration hindered balanced improvement of environmental benefits across the prefectural cities.

## 5. Conclusions

This study analyzed urban environmental benefits among China’s major cities based on their structure of urban land use in 2000 and 2010. We developed an assessment index system covering three aspects of environmental benefits, including green infrastructure, industrial upgrade, and urban environmental management. Our results showed that higher income and urban population growth did bring about improvement of the environmental benefits in all cities in 2000–2010; however, coastal and capital cities had less improvement on average than the other cities. We also found that improvement of the environmental benefits was mostly dependent on green infrastructure expansion, with the least contribution from industrial upgrade. This pattern was particularly conspicuous among *RD* cities.

As urban expansion continues at a rapid rate, it is important for China to promote balanced improvement of environmental benefits. First, the environmental benefits among green infrastructure improvement, industrial upgrade and environmental management improvement should be balanced. Specifically, more attention should be paid to industrial upgrade and better environmental management (e.g., harmless treated domestic garbage, management of sewerage, landscaping, and environmental sanitation). Second, environmental improvement between *RD* and *RP* cities should be balanced through regional differentiation policies. For *RD* cities, it is urgent to promote industrial upgrade and transformation to a service economy for improving the environmental benefits in the long run. For *RP* cities, it is urgent to increase income level and urban population growth through the promotion of high value-added industries, some of which could be transferred from *RD* cities to alleviate their demographic agglomeration. At the same time, more urban land quota could be arranged in *RP* cities through land use planning to reduce urban land agglomeration in *RD* cities. Looking to the future, we suggest that urban land use needs to be integrated into rural-urban interactions so that viable and sustainable pathways can be developed for flows of resources, goods and people under changing environmental and economic conditions.

## Figures and Tables

**Table 1:** Assessment index system of environmental benefits of urban land use.

Standard Layer	Index	Sign	Definition	Unit
Green infrastructure	Area of parks and green space per capita	+	The ratio of area of parks and green space to urban population	m^2^
	Density of parks and green space	+	The ratio of area of parks and green space to area of built district	%
	Park size	+	The ratio of park area to number of parks	hm^2^
Industrial upgrade	Density of industrial land	-	The ratio of industrial land area to urban construction land area	%
	Density of industrial SO_2_ discharged	-	The ratio of volume of industrial SO_2_ discharged to urban construction land area	t/km^2^
	Rate of discharged industrial wastewater meeting national standard	+	The ratio of volume of discharged industrial wastewater meeting national standard to volume of industrial wastewater discharged	%
Environmental management	Proportion of urban maintenance and construction fund invested in environmental expenditure	+	The ratio of expenditure fund for sewerage, landscaping and environmental sanitation to the total expenditure of urban maintenance and construction fund	%
	Intensity of urban maintenance and construction fund invested in environmental expenditure	+	The ratio of expenditure fund for sewerage, landscaping and environmental sanitation to urban construction land area	10^4^ yuan per km^2^
	Disposal rate of domestic harmless garbage	+	The ratio of volume of domestic garbage harmless treated to volume of domestic garbage treated	%

Data sources: Volume of industrial SO_2_ discharged, volume of industrial wastewater discharged meeting national standard, and volume of industrial wastewater discharged were obtained from *China City Statistical Yearbook*; urban population and total population were obtained from *Tabulation on The Population Census of The People’s Republic of China*; per capita disposable income of urban households was obtained from *China Statistical Yearbook for Regional Economy*, and corrected by price index; and all the other data were obtained from *China Urban Construction Statistical Yearbook*. “+” means value of the index is positively related to environmental benefits of urban land use, and vice versa.

**Table 2:** Definition of independent variables.

Variable		Definition	Unit
Per capita income	*pci*	Per capita disposable income of urban households	10^4^ yuan
Urban population	*aup*	Size of urban population	10^4^
Per capita urban construction land area	*pcucl*	The ratio of urban construction land area to amount of urban population	m^2^
Demographic urbanization	*du*	The ratio of amount of urban population to total amount of population	%
Land urbanization	*lu*	The ratio of urban construction land area to total land area	%

Data sources: *pci* was obtained from *China Statistical Yearbook for Regional Economy* was corrected by price index; other variables were obtained from the same data sources as shown in Table 1.

**Table 3:** Change in environmental benefits of urban land use, 2000–2010.

Cities	Number	Average Increase				Contribution (%)		
		*ebu*	*ebugi*	*ebudi*	*ebuma*	*ebugi*	*ebudi*	*ebuma*
The whole prefectural cities	347	0.638	0.279	0.151	0.208	43.682	23.692	32.626
The *RD* cities	81	0.423	0.260	0.071	0.092	61.584	16.775	21.641
The *RP* cities	266	0.703	0.284	0.175	0.244	40.403	24.958	34.638

*Note: ebu* denotes environmental benefits of urban land use; *ebugi* denotes environmental benefits of green infrastructure; *ebudi* denotes environmental benefits from industrial upgrade; *ebuma* denotes environmental benefits from environmental management.

**Table 4:** Change in environmental benefits of urban land use in Changzhou and Jiujiang, 2000–2010.

Cities	*ebu*		Increase				Contribution (%)		
	2000	2010	*ebu*	*ebugi*	*ebudi*	*ebuma*	*ebugi*	*ebudi*	*ebuma*
Changzhou	4.90	5.35	0.46	0.27	0.03	0.15	59.20	7.21	33.59
Jiujiang	4.62	5.99	1.37	0.68	0.14	0.55	49.64	10.22	40.15

*Note: ebu* denotes environmental benefits of urban land use; *ebugi* denotes environmental benefits of green infrastructure; *ebudi* denotes environmental benefits from industrial upgrade; *ebuma* denotes environmental benefits from environmental management.

**Table 5:** The original values of assessment indices of environmental benefits of urban land use, Changzhou and Jiujiang.

Standard Layer	Index	Changzhou		Jiujiang	
		2000	2010	2000	2010
Green infrastructure	Area of parks and green space per capita (m^2^)	19.19	28.54	28.08	74.53
	Density of parks and green space (%)	28.34	41.92	28.55	54.35
	Park size (hm^2^)	8.46	21.56	24.78	41.13
Industrial upgrade	Density of industrial land (%)	32.58	23.84	24.89	24.01
	Density of industrial SO_2_ discharged (t/km^2^)	56.10	14.24	62.50	3.86
	Rate of discharged industrial wastewater meeting national standard (%)	96.40	99.98	71.10	92.05
Environmental management	Proportion of urban maintenance and construction fund invested in environmental expenditure (%)	21.37	26.94	23.14	21.93
	Intensity of urban maintenance and construction fund invested in environmental expenditure (10^4^ yuan per km^2^)	2.92	4.76	0.58	12.81
	Disposal rate of domestic harmless garbage (%)	84.05	98.79	39.11	79.24

**Table 6:** Statistical description of variables in 2000 and 2010.

		*ln(ebu)*	*ln(pci)*	*ln(aup)*	*ln(pcucl)*	*ln(du)*	*ln(lu)*
2000	Mean	1.54	8.63	4.03	4.30	3.91	−1.01
	Median	1.55	8.59	4.06	4.28	3.94	−0.79
	Maximum	1.85	9.98	7.26	6.02	4.61	3.18
	Minimum	1.25	8.08	0.82	1.98	2.21	−5.31
	Std. Dev.	0.08	0.27	1.10	0.45	0.43	1.44
	Observations	347	347	347	347	347	347
2010	Mean	1.67	9.69	4.31	4.59	4.07	−0.47
	Median	1.67	9.65	4.31	4.56	4.08	−0.36
	Maximum	1.95	10.48	7.61	6.31	4.61	3.71
	Minimum	1.46	9.02	1.25	3.20	3.13	−4.74
	Std. Dev.	0.07	0.23	1.09	0.37	0.30	1.47
	Observations	347	347	347	347	347	347

**Table 7:** Regression result of change in environmental benefits of urban land use.

Variable	2000			2010		
	Coefficient	Std. Error	t-Statistic	Coefficient	Std. Error	t-Statistic
Constant	−0.062	0.030	−2.046	−0.621	0.033	−18.932
*ln*(*pci*)	0.161	0.004	44.744	0.213	0.003	61.873
*ln*(*aup*)	0.076	0.008	9.506	0.303	0.005	55.644
*ln*(*pcucl*)	−0.008	0.001	−16.041	0.020	0.001	21.401
*ln*(*du*)	0.053	0.001	42.810	0.051	0	137.783
*ln*(*lu*)	−0.002	0	−5.457	−0.009	0	−48.689
*ln*(*pci*)×*ln*(*aup*)	−0.008	0.001	−8.610	−0.033	0.001	−52.164
*R^2^*	0.995			*R^2^*	0.994	

*Note*: The independent variables are all significant at the 0.05 level.
